# Voiding cystourethrography for the pediatric nephrologist: clinical value, challenges, and areas of debate

**DOI:** 10.1007/s00467-025-06901-3

**Published:** 2025-09-09

**Authors:** Pierluigi Marzuillo, Angela La Manna, Pier Luigi Palma, Paola Tirelli, Anna Di Sessa, Anna Russo, Alfonso Reginelli, Agnese Roberti, Laura Verde, Stefano Guarino, Giovanni Di Iorio

**Affiliations:** 1 Department of Woman, Child and of General and Specialized Surgery, Università Degli Studi Della Campania “Luigi Vanvitelli”, Via Luigi De Crecchio 2, Naples, Italy; 2 Radiology Unit, Department of Precision Medicine, Università Degli Studi Della Campania “Luigi Vanvitelli”, Naples, Italy; 3 Pediatric Urology Unit, “Santobono-Pausilipon” Children’s Hospital, Naples, Italy; 4 Department of Mathematics and Physics, Università Degli Studi Della Campania “Luigi Vanvitelli”, Caserta, Italy

**Keywords:** Voiding cystourethrography, Vesicoureteral reflux, Posterior urethral valves, Urinary tract infection

## Abstract

Voiding cystourethrography (VCUG) is indicated for evaluating the anatomy of the urethra and bladder. It is primarily used to diagnose vesicoureteral reflux (VUR) and posterior urethral valves (PUV), especially in cases of febrile urinary tract infections (UTIs), significant ureteral dilation on ultrasound, and—in continent children—urinary symptoms suggestive of infravesical obstruction. VCUG exposes children to a non-negligible dose of radiation and requires urethral catheterization, which can be painful—particularly in males—and carries a low but present risk of post-procedural UTI. Despite its widespread use, VCUG can miss VUR in up to 50% of cases. Moreover, recent studies report that preoperative suspicion of PUV on VCUG was present in only 46% of non-toilet-trained and 59% of toilet-trained males, likely due to challenges in interpreting the VCUG findings. In this review, we will highlight the clinical value, challenges, and areas of debate surrounding this diagnostic test, and offer practical tips to optimize its performance.

**Graphical abstract:**

**Figure:**
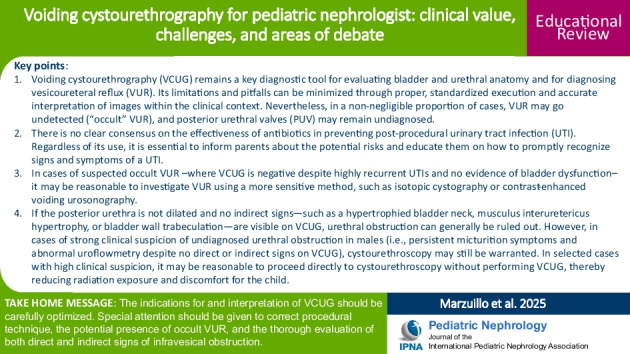
A higher resolution version of the Graphical abstract is available as Supplementary information

**Supplementary Information:**

The online version contains supplementary material available at 10.1007/s00467-025-06901-3.

## Introduction

Voiding cystourethrography (VCUG) is a radiological procedure that uses X-rays and is considered the gold standard for assessing bladder and urethra anatomy, as well as for diagnosing vesicoureteral reflux (VUR) [1, 2].

However, VCUG is an invasive procedure associated with both economic and radiation-related costs. It requires urethral catheterization, which can be painful—especially in males—and may be complicated by post-procedural fUTI in 1–12% of cases, particularly in children with dilated VUR, even when receiving antibiotic prophylaxis [3–6].

VCUG also has important limitations, which call for careful consideration of its indications and cautious interpretation of the images. Notably, it can fail to detect VUR and posterior urethral valves (PUV) in up to 50% of cases [7–12].

In this review, we will discuss the clinical value, challenges, and areas of debate surrounding this diagnostic tool. Additionally, we offer practical tips to optimize its diagnostic performance for detecting VUR and PUV.

## Voiding cystourethrography procedure: good practice (summarized in Box 1)

Box 1 VCUG: good practice

**Table:** 

**PREPARATION**
- Without sedation or immobilization but in a comfortable environment and in presence of parent/s
- In toilet-trained children bladder emptying with urination in the bathroom, in non-toilet-trained children at the time of catheterization
- Urine dipstick test
- Clean washing of the genital area
**CATHETERIZATION**
- Sterile method
- Intraurethral application of lubricant anesthetic
- Transurethral positioning in bladder of nasogastric tube previously lubricated, with size appropriate for age (5–8Fr), of adequate length to avoid loops, and externally fixed to skin with adhesive paper (plaster)
**CONTRAST MEDIUM INFUSION**
- Bottle 50–100 cm above the table, gravity dripping, until spontaneous urination
- Cyclical (2–3) filling up to age of 1–2 years
**IMAGING**
- Follow ALARA and Image Gently principles
- Fluoroscopic scout images (to reduce radiation images should be saved from fluoroscopy rather than obtained by further exposures)
- Before bladder filling AP image
- At early and late filling AP and oblique images
- At voiding phase images of the urethra, AP in females, oblique in males
- Post-void image AP for bladder residual and, if VUR, for contrast drainage from the ureters
**TIMING OF VCUG AFTER UTI**
- Irrelevant to the detection of VUR
**POST-PROCEDURAL UTI**
- Acquire information on the presence of dilated VUR
- Monitoring and education of parents to recognize the appearance of suspicious symptoms for UTI
- Possibly antibiotics in high-risk patients (dilated VUR)

*Abbreviations: ALARA*, as low as reasonably achievable; *AP*, anteroposterior; *VCUG*, voiding cystourethrography; *UTI*, urinary tract infection; *VUR*, vesicoureteral reflux.

When performing a VCUG, technical errors—such as insufficient bladder filling, performing only a single fill-and-void cycle in infants, or failure to visualize the urethra during the voiding phase—can lead to inaccurate results.

To standardize VCUG performance, the American Academy of Pediatrics published a protocol in 2016 emphasizing the ALARA (“as low as reasonably achievable”) and Image Gently principles. According to these, pediatric radiologists must minimize radiation exposure as much as possible while still achieving diagnostic utility [1]. Similarly, in 2024, the European Society of Pediatric Radiology published updated recommendations for VCUG [2].

VCUG should be performed only when the patient is asymptomatic and has a negative urine dipstick test at the time of catheterization. Prior to the procedure, informed consent must be obtained from the parents or legal guardians.

When conducting a VCUG, special attention should be paid to the anxiety and pain of the child. A parent should remain with the child throughout the procedure. Sedation may be used, provided it does not interfere with voiding dynamics or compromise diagnostic accuracy [1], although it is generally not required [2]. Topical anesthetics, such as lidocaine gel, may help reduce discomfort [2].

Nonetheless, the absence of sedation may lead to motion artifacts, suboptimal image quality, or even the need to repeat the examination. Although catheterization and the instillation of lubricant are generally tolerable, they can still cause variable degrees of pain. Given the minimal biological impact and proven safety of intranasal—or preferably oral—midazolam, and its lack of effect on voiding or VUR detection [13], the use of procedural sedation could be considered in clinical practice.

Prior to endourethral anesthetics instillation and catheterization, the genital area should be thoroughly cleaned. The use of antiseptics remains controversial, as studies suggest they do not significantly reduce the risk of post-procedural infections [2, 14, 15]. At our center, cleansing is performed using sterile gauze and 0.9% saline solution, without antiseptics.

Although we do not routinely use sedation, we focus on creating a calm environment and applying adequate topical anesthesia using a lidocaine-containing lubricant. This approach significantly reduces discomfort and is recommended in children [16].

Toilet-trained children should void in a private restroom before the examination. In non-toilet-trained patients, the bladder is emptied at the time of catheterization.

In males, lidocaine is instilled directly into the urethra using a sterile syringe without the needle or with a specific, more expensive device designed for this purpose. In females, lidocaine can be applied to the interlabial area (especially in cases of local genital hyperaemia), followed by intraurethral gel instillation. However, given the short length of the female urethra, our experience suggests that catheterization is only minimally painful, and the use of a lubricated catheter alone is often sufficient.

Importantly, the analgesic effect of lidocaine-containing lubricants is immediate, and waiting after instillation is unnecessary. A study by Poonai et al. demonstrated that lubrication itself is more effective for pain relief than lidocaine, which can be associated with increased pain during instillation [17]. Based on our clinical experience, this is more commonly perceived as mild discomfort—occasionally reported as a tingling sensation by older children—rather than as true pain.

To further reduce the risk of contamination and post-procedural UTIs, we suggest using a new, single-use tube for each patient, especially when specific disposable devices are not available. Reusing the same tube for multiple patients on different days should be avoided.

Bladder catheterization should always be performed by trained personnel. In uncircumcised male patients, particular care should be taken to reposition the foreskin after retraction during the procedure to avoid the risk of paraphimosis [2].

At our center, we use a sterile 5- or 6-Fr nasogastric tube as a non-balloon catheter, ensuring proper sterile technique (handwashing, sterile field, gloves). Adjusting catheter length is critical to prevent the formation of loops within the bladder.

**Fig. 1:**
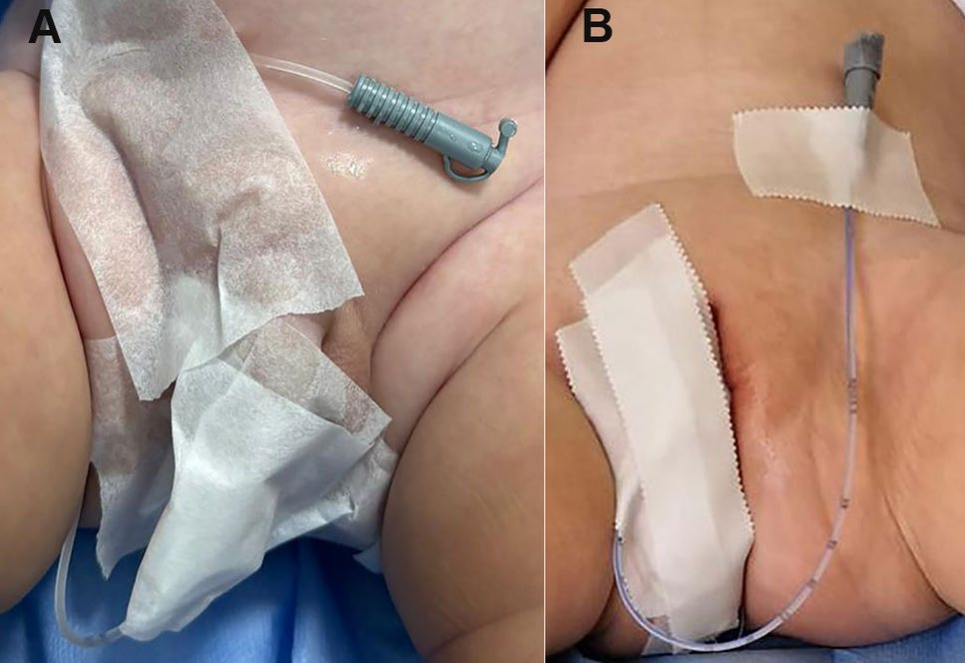
Fixation of a CH 5 nasogastric tube in a male (**A**) and a female (**B**)

The nasogastric tube is fixed as illustrated in Fig. 1. As suggested by Damasio et al. [2], we prefer nasogastric tubes over balloon catheters because they are smaller and allow a second fill-and-void cycle without the need to remove and reinsert the catheter.

However, in the absence of a nasogastric tube, a Foley catheter may be used without inflating the balloon, securing it in place as shown in Fig. 1. Leaving the balloon uninflated, rather than deflating it after bladder filling, may help avoid missing the voiding phase while the operator deflates the balloon at the radiology table.

In males, an oblique or lateral projection is recommended for better visualization of the urethra, ideally including at least one image without the catheter [2]. Nonetheless, if the nasogastric tube is well secured (as shown in Fig. 1), it may remain in place during the voiding phase, ensuring adequate urethral visualization. Although adhesive contamination with contrast medium may occur after the first voiding, it rarely compromises the quality of images during the second filling phase.

The European recommendations recommend immobilization devices, particularly in non-cooperative children [2]. These help minimize radiation exposure by reducing motion artifacts and avoiding repeat examinations [2].

A single anterior–posterior (AP) scout image should be taken, and it should include kidneys, ureters, and bladder. The bladder is then filled with contrast agents such as iothalamate meglumine or full-strength diatrizoate meglumine. Images should be acquired in AP, right and left oblique, and lateral projections. Warming the contrast medium to near body temperature may improve patient comfort. Bladder filling should be slow—especially in young children—to facilitate the detection of ureteroceles (about 10% of expected bladder capacity per minute). The expected bladder capacity is calculated as weight (kg) × 7 for patients < 2 years, (30 × age in years) + 30 in patients > 2 to 14 years, and 500 ml in patients > 14 years of age [1, 2].

However, when using a nasogastric tube, we typically fill the bladder until the child expresses the need to void (toilet-trained) or voiding occurs spontaneously (non-toilet-trained). Thus, expected bladder capacity is used primarily for interpretation purposes, to assess deviations that may suggest underlying voiding dysfunction.

Patients with volumes significantly below or above expected capacity may have filling or voiding disorders, respectively. Including this information in the report may increase the diagnostic utility of VCUG.

During voiding phase, images including entire urethra from the bladder neck to the tip of the penis with lateral or oblique projections in males, and AP urethra images in girls should be taken.

One final image of the bladder, ureters, and kidneys is taken within 5 min after voiding to conclude the study. Post-void bladder imaging is essential for evaluating post-void residual volume (normal if highly refluxing ureters empty into the bladder after micturition) and for assessing whether the refluxing ureters empty completely into the bladder. The persistence of contrast in the ureters after micturition helps identify a concomitant obstructive component associated with VUR.

After documenting the urethra during micturition, toilet-trained patients who are unable to completely empty their bladder (due to overfilling or embarrassment) may be allowed to urinate in the restroom. In such cases, post-void images should be acquired within 5 min.

As recommended [1, 2], images should be saved from the fluoroscopic imaging, rather than from additional exposures, to minimize radiation.

VUR should be graded using the International Reflux Grading System [18]. Studies show considerable interobserver variability, particularly between grades II and IV [19–21]. However, if the clinical management of VUR is guided by symptoms, in our opinion, this variability is unlikely to impact appropriate patient management.

**Fig. 2:**
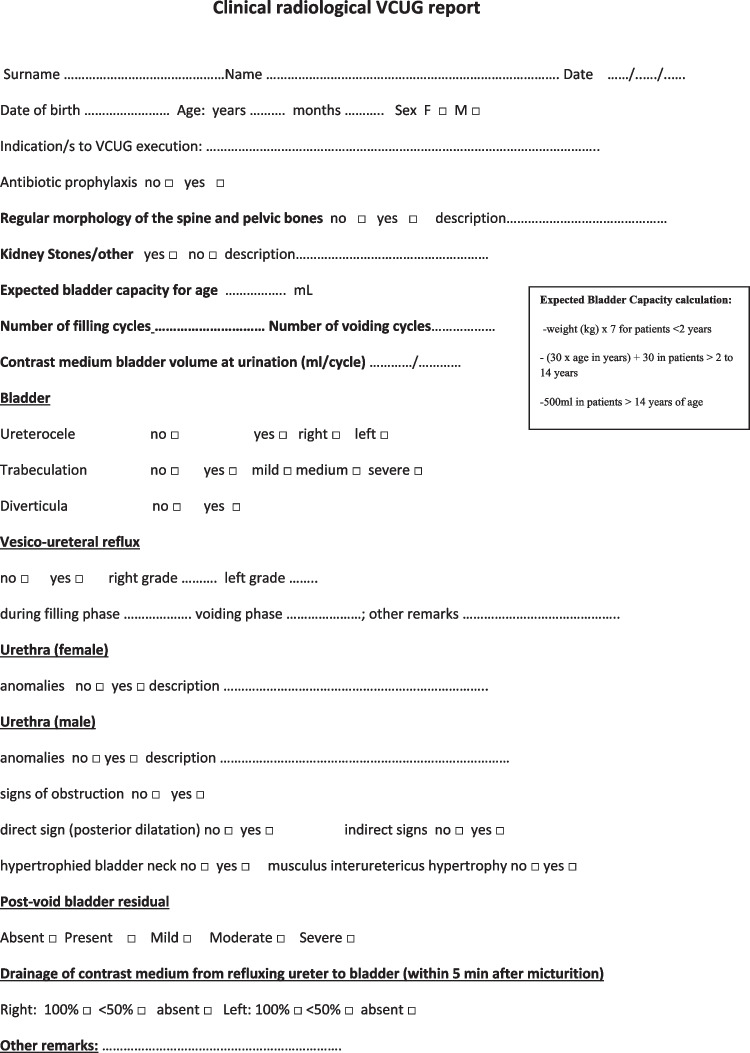
Clinical radiological VCUG report

Finally, it is important to develop a structured report for VCUG [2]. The implementation of a structured report may enhance the consistency and accuracy of the recorded information, while also facilitating standardized data collection and enabling meaningful comparisons across different institutions [22]. Damasio et al. [2] proposed a structured report. Here, we present the model adopted at our center for this type of report (Fig. 2).

## Current indications to voiding cystourethrography

### Urinary tract infections

Among congenital anomalies of the kidney and urinary tract (CAKUT), VUR is the most frequently associated with fUTIs [23], since 25–40% of children presenting fUTIs are found to have VUR [24]. To date, VCUG is used by clinicians to detect VUR in order to prevent fUTIs [24], and potential subsequent renal scar formation [25, 26]. After a single fUTI, different diagnostic approaches are recommended based on bacterial etiology of the infection and/or any kidney and urinary tract anomalies detected on ultrasound. A kidney ultrasound (KUS) can be performed after the acute phase of fUTI, typically 4–6 weeks later. The presence of specific abnormalities (unilateral or bilateral kidney hypoplasia, anomalies of parenchymal echogenicity, pelvi-calyceal dilation ≥ second degree according to Society of Fetal Urology, ureteral dilation, thickening of the renal pelvis and, bladder abnormalities) or a fUTI caused by non-*E. coli* bacteria allows clinicians to perform VCUG [27–30]. Additionally, VCUG is indicated if two or more fUTIs due to *E. coli* or a complicated/atypical fUTI (i.e., abscess formation) occur [27–32].

In addition, performing a VCUG may be reasonable in cases of recurrent non-febrile UTIs in male children, especially if associated with abnormal ultrasound findings (such as reduced or increased bladder volume, thickening of the bladder wall, significant post-void residual urine, or diverticula) or with micturition symptoms [33] in order to rule out infravesical obstruction.

### Urinary tract dilation

Dilation of the urinary tract remains a significant clinical challenge in deciding which patient will benefit from antibiotic prophylaxis, urological diagnostics, or treatment [34].

The widespread use of KUS during pregnancy has resulted in a higher detection rate for antenatal hydronephrosis. VCUG may be performed after postnatal confirmation of prenatal dilation or following any postnatal KUS showing one or more of the following: mono- or bilateral megaureter > 7 mm in diameter, mono- or bilateral hydronephrosis with APDP > 15 mm, small kidney (kidney length < 2 standard deviation score) and/or mono/bilateral renal dysplasia (cortical thinning, poor corticomedullary differentiation, renal cysts) [35]. According to the latest urinary tract dilation classification, VCUG may also be recommended when the APDP is ≥ 10 mm, in the presence of peripheral calyceal dilatation or a ureter measuring ≥ 4 mm, or in cases with associated bladder or renal parenchymal anomalies, or oligohydramnios [36].

Some of these indications remain a matter of debate—for example, whether to perform a VCUG in cases of severe unilateral pelvicalyceal dilation (more suggestive of obstructive pathology) or mild dilation without a history of fUTIs. In any case, clinical manifestations such as UTIs and micturition symptoms should be carefully considered when evaluating the indication for VCUG in children with urinary tract dilation.

### Other indications

Micturition symptoms and uroflowmetry findings suggestive of infravesical obstruction represent further indications to VCUG [33]. More specifically, VCUG is indicated to study the urethra in males, even without fUTIs, in case of persistent micturition symptoms (daytime incontinence and/or straining to void and/or hesitancy and/or urgency and/or frequency with/without nocturnal enuresis, urinary retention or a reduced number of daily voidings), low Qmax on repeated uroflowmetries, non-febrile UTI (especially if recurrent), and/or ultrasound findings (such as thickening of bladder wall, post-void residual, bladder wall trabeculation, bladder diverticula, unilateral or bilateral megaureter, etc.) that suggest lower urinary tract obstruction [33, 37]. The combination of uroflowmetry followed by ultrasound evaluation of post-void residual represents the non-invasive urodynamic.

VCUG is also useful for evaluating complex conditions, including neurogenic bladder and urinary tract anomalies associated with syndromes such as imperforate anus, urogenital sinus, and prune belly syndrome [2].

In patients with neurological lower urinary tract dysfunction, an alternative to conventional VCUG is the videourodynamic study, which combines a urodynamic assessment with VCUG to provide information on bladder and urethral sphincter function, in addition to the anatomical details typically obtained from VCUG, all through a single bladder catheterization [38]. Furthermore, Rosen et al. demonstrated that radiation exposure from the videourodynamic study was lower than that of VCUG across all age groups [38].

Additionally, VCUG is indicated for the follow-up of previously diagnosed VUR [35], although this is limited to a few selected cases—primarily when surgical treatment of reflux is being considered, in the event of fUTIs after surgery, and always after confirming and addressing any underlying bladder dysfunction. Repetition of VCUG is not indicated after surgery. The need for follow-up VCUG after endoscopic surgery remains a matter of debate; some authors recommend a control study 3–12 months after the procedure, while it appears more reasonable imaging only in the case of a subsequent fUTI [2, 39–41].

## Timing of VCUG execution post-UTI

Traditionally, there have been concerns regarding the accuracy and safety of performing a VCUG during an active infection. In the past, it was hypothesized that UTIs could cause ureteral dilation and inflammatory changes at the ureterovesical junction, potentially resulting in transient VUR or its overestimation [42]. However, McDonald et al. demonstrated that in hospitalized children who underwent VCUG within a week of UTI diagnosis, the prevalence of reflux does not differ significantly from those examined at a later time [43]. Additionally, there are anecdotal concerns that if performing VCUG during an active UTI, the catheterization might damage the already inflamed urinary tract mucosa, potentially prolonging the UTI or causing bacterial dissemination and sepsis [44, 45]. However, evidence suggests that 75% of patients with fUTI achieve urine sterilization within just 9 h [46]. Therefore, if a VCUG is performed during the course of a fUTI after initiating appropriate antibiotic treatment and observing a clinical response, it is unlikely to prolong the infection or cause bacterial dissemination or sepsis.

In conclusion, reassuring evidence indicates that the prevalence and severity of VUR in children with UTI are not influenced by the timing of VCUG execution [43, 47–49] and that the risk of secondary infection, bacterial dissemination, or urosepsis is irrelevant [45, 49]. Therefore, VCUG can be performed at any time after completing UTI treatment [29]. However, considering this evidence and for local resource optimization, in our opinion, it can also be performed during hospitalization for the fUTI episode if there are already indications to proceed with VCUG [27–32].

## Post-procedural urinary tract infections

VCUG is an invasive procedure whose possible complications are due to the positioning of the bladder catheter. Most complications—such as urethral trauma, improper catheter placement, or bladder perforation—are rare or anecdotal in experienced hands. The most common complication is post-procedural UTI, although its incidence varies [50]. A post-procedural UTI can reasonably be defined as the onset of symptoms within 5 days following the VCUG. As described above, it is essential to perform the procedure in a sterile manner. Furthermore, antibiotic prophylaxis may be useful in preventing post-procedural UTI. The most recent studies on post-procedural UTIs are summarized in Table 1. The incidence of post-procedural UTI ranges from 1 to 12%. Risk factors for post-procedural UTI include pre-existing urological conditions. However, when specified in the studies, the primary risk factor is the presence of dilated VUR at the time of VCUG [3–6, 51–53].

**Table 1:** Studies on post-VCUG UTIs in children

Study (year)	Design of the study	Number of VCUG	Definition of post-VCUG UTIs	Number (%) of post-VCUG UTIs	Antibiotic scheme	Risk factors for post-VCUG UTIs	Number (%) of patients on antibiotic prophylaxis	Antibiotic prophylaxis recommended
Rachmiel et al. (2005)	Retrospective	421	Symptoms of UTIs (febrile or not), and positive urine culture within 7 days post-VCUG	5 (1.2%)	Patients received cephalexin 20 mg/kg once daily as baseline prophylaxis, increased to 50 mg/kg/day in three divided doses the day before, the day of, and the day after VCUG, then returned to 20 mg/kg once daily	Presence of vesicoureteral reflux with higher degree increasing the risk	421 (100%)	Yes
Moorani et al. (2010)	Retrospective	100	Symptoms of UTIs (febrile or not), and positive urine culture by sterile-bag or mid-stream urine within 10 days post-VCUG	12 (12%)	Not specified	Presence of congenital urinary tract anomalies	Not specified	Yes
Johnson et al. (2017)	Retrospective	1203	Symptoms of UTIs (febrile or not), positive urinalysis, and positive urine culture within 7 days post-VCUG	12 (1.0%)	33% of patients on prophylactic dose, 14.5% on therapeutic dose, 2.9% unknown dosing	Preexisting urological diagnosis, abnormal VCUG results, use of periprocedural antibiotics	607 (50.5%)	No
Sinha et al. (2017)	Randomized control trial	120	Symptoms of UTIs (febrile or not) by catheterization or mid-stream urine within 3 days post-VCUG	9 (7.5%)	Patients were randomized in a 3:2 ratio to either the antibiotic group or the non-antibiotic group. In the antibiotic group, cephalexin (10–15 mg/kg three times daily) was administered to infants under 6 months of age, and co-trimoxazole (4 mg/kg twice daily) to those older than 6 months. Nitrofurantoin was used in patients with a history of hypersensitivity to these antibiotics. Antibiotics were prescribed for three days: the day before, the day of, and the day after the VCUG	Not undergoing antibiotic prophylaxis, preexisting abnormal US anomalies	72 (60%)	Yes
Marzuillo et al. (2019)	Retrospective	216	Urinary leukocytes and/or nitrites, positive urine culture, and fever > 38 °C, within 7 days post-VCUG	5 (2.3%)	Within 15 min after the end of the VCUG, the protocol of antibiotic administration was the following: (i) if VUR was not present or a non-dilated VUR was detected, no antibiotics; (ii) if dilated VUR was present, 5 mg/kg of intramuscular netilmicin followed by oral amoxicillin clavulanate at the dose of 25 mg/kg every 12 h for 5 days	Presence of dilated or bilateral VUR	76 (35.2%)	Yes if dilated VUR
Martins et al. (2020)	Retrospective	513	Symptoms of UTIs (febrile or not), and positive urine culture by suprapubic aspiration or catheterization or mid-stream urine within 7 days post-VCUG	23 (4.5%)	The specific dosage was not reported; however, 95.5% of patients received trimethoprim alone, 2.6% received nitrofurantoin, 1.2% received amoxicillin-clavulanic acid, and 0.7% received co-trimoxazole	Presence of VUR	268 (52.2%)	No
Doval et al. (2024)	Retrospective	318	Fever and positive urine culture by catheter within 7 days post-VCUG	12 (3.8%)	Not specified	No risk factors were identified	107 (33.6%)	Not specified

*AAP*, American Academy of Pediatrics; *US*, ultrasound; *UTI*, urinary tract infection; *VCUG*, voiding cystourethrography; *VUR*, vesico-ureteral reflux

All studies were retrospective in design, except for the randomized controlled trial by Sinha et al. [4], which demonstrated better outcomes in children who received periprocedural antibiotic prophylaxis. Based on the available evidence, there is no consensus regarding the indications for antibiotic prophylaxis, the choice of antibiotic, the timing of initiation, dosage, and duration of treatment (Table 1). It may be reasonable to prescribe antibiotics only for patients with dilated VUR detected on VCUG, starting antibiotics just after the VCUG to prevent fUTI. This implies the need to know in real time whether dilated VUR is present on VCUG. However, it is generally agreed in the studies that the best method to prevent post-procedural UTI is the use of the cleanest catheterization technique possible [3–6, 51–53].

Regardless of the decision to prescribe antibiotic prophylaxis when submitting a child to VCUG, it is important to instruct parents to recognize post-cystography UTI by performing a urine test if there is a fever or urinary symptoms within 5 days of the procedure, in order to promptly diagnose a UTI.

## VCUG for VUR diagnosis: areas of debate

As previously noted, VCUG is widely used to detect VUR, but not all VUR cases are the same. Two entities have been proposed: VUR as a “disease” and VUR as a “symptom” [54]. The first type, VUR as a disease, is usually high grade (IV–V) and primarily affects male infants. It is usually characterized by abnormal kidney parenchyma, urinary tract dilation, and a low rate of spontaneous resolution. The second type, VUR as a symptom, is usually low grade (I–III) and primarily affects female children at a later age. These children typically have normal kidney parenchyma, no urinary tract dilation, and a high rate of spontaneous resolution [54]. Therefore, it is legitimate to question whether it is worth diagnosing the second type of VUR. Considering the benignity of the VUR as a “symptom,” a more selective approach could be considered. Excluding cases where the clinical and instrumental presentation suggests obstructive uropathy requiring a specific approach [34] or where abnormalities of kidney parenchyma are clearly evident [54], we wonder if the indications for VCUG solely for VUR detection could be limited to cases where clinical intervention could result from this radiological technique (e.g., surgical correction of the most severe VUR cases presenting with recurrent fUTIs).

VUR is often investigated with the aim of preventing fUTI episodes through continuous antibiotic prophylaxis. However, the decision to submit a patient to continuous antibiotic prophylaxis for a VUR depends more on the frequency of fUTIs, age, gender, and possible concomitant bladder dysfunction than on VUR itself, even in cases of dilated VUR. This is particularly relevant since the change in the number of focal defects (new kidney scarring) on Tc99m dimercaptosuccinic acid scintigraphy was similar in children with and without continuous antibiotic prophylaxis and was independent of the occurrence of fUTIs [55]. This could support our recommendation to investigate VUR only in cases where its diagnosis could lead to clinical intervention.

Moreover, in females, where information about urethral morphology is not needed, techniques with continuous monitoring of the bladder filling and voiding, such as isotopic cystography (IC) and contrast-enhanced voiding urosonography (ceVUS), may be preferred for detecting VUR [10, 56]. Additionally, this latter technique has the advantage of being radiation-free [50].

Finally, even when correctly performed, VCUG can miss the diagnosis of VUR in up to 50% of the cases (“occult” VUR), which can instead be detected by IC [7–10]. The “occult” VUR could be of severe degree in approximately 70% of cases, with scintigraphic damage evident in about 50% of them. These patients may also have a significant risk of developing fUTIs, proteinuria, reduced kidney function (estimated glomerular filtration rate < 90 mL/min/1.73 m^2^), or hypertension, necessitating long-term follow-up. Therefore, if a patient undergoes VCUG for a strong suspicion of VUR (recurrent fUTIs) but does not show VUR, it might be reasonable to search for VUR with techniques involving continuous monitoring of the bladder filling and voiding, such as IC because an “occult” VUR, potentially requiring surgical correction, could be present [10].

As an alternative—though invasive—method for detecting occult VUR, Positional Instillation of Contrast Cystography (PIC) may be considered [57]. In their systematic review, Pakkasjärvi et al. reported that among 496 symptomatic patients (with recurrent febrile UTIs or febrile UTIs despite antibiotic prophylaxis), with a mean age of 6.8 years and negative VCUG, PIC identified occult VUR in 73% of cases [57].

The procedure involves positioning the cystoscope in front of the ureteric orifice in an empty bladder and instilling contrast directly at the orifice through the irrigation port of the cystoscope [57]. Due to its invasiveness, we recommend cautious use of PIC, reserving it for patients with recurrent febrile UTIs and a strong clinical suspicion of occult VUR, and when there is the possibility of performing endoscopic treatment of reflux during the same session, if present. In light of this, the goal of clinicians should not be to diagnose VUR at all costs but rather to accurately interpret clinical signs in order to optimize VUR diagnostics as effectively as possible.

CeVUS has emerged as an alternative method for the evaluation of VUR. This technique demonstrates high diagnostic performance, with reported sensitivity and specificity rates of 90% and 92.8%, respectively [58].

## VCUG for PUV diagnosis: areas of debate

PUV are the leading cause of lower urinary tract obstruction in boys [59]. They account for 17% of cases of stage 5 chronic kidney disease in children and can also lead to voiding symptoms in older children [11]. Most cases are detected during pregnancy or just after birth (“neonatal” PUV) [59, 60]. However, about one-third of patients may receive a delayed diagnosis (after one year of age) (“late” PUV) when micturition symptoms, with or more frequently without UTIs, appear [61, 62]. The variability in PUV incidence may result from the absence or weakness of the clinical and/or radiological reference standard [12].

In the presence of suspicious signs or symptoms, although a definitive diagnosis of PUV is endoscopic [59], urethrocystoscopy is usually preceded by a VCUG, as it is considered the radiological gold standard [63].

**Fig. 3:**
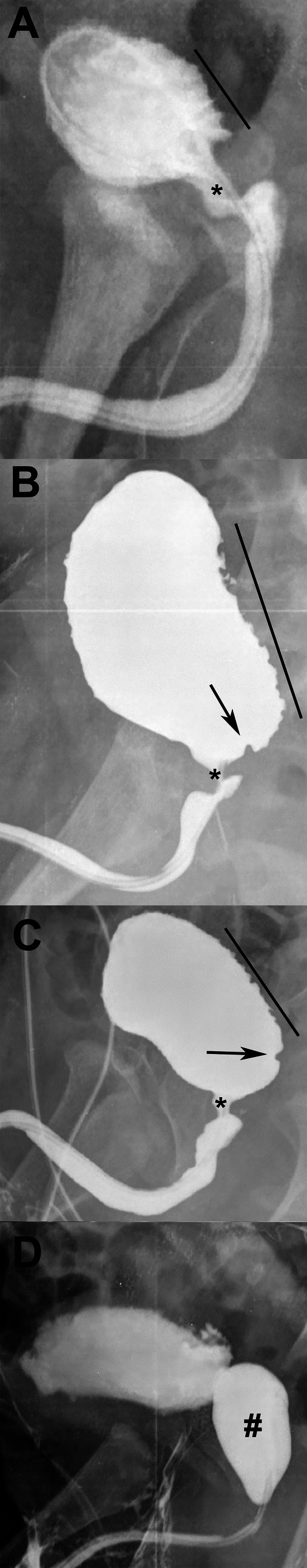
Direct and indirect signs of PUV. Indirect signs of PUV are indicated by a hypertrophied bladder neck (unusual indentation of the bladder neck on lateral/oblique images during voiding, marked by an asterisk in **A**–**C)**, musculus interuretericus hypertrophy (indentation at the level of the ureteral orifices on lateral/oblique images during bladder filling or voiding, marked by an arrow in **B**–**C**), and a trabeculated appearance of the bladder wall (toothed aspect of the bladder wall, indicating detrusor muscle hypertrophy, concerning the pars fixa dorsally during filling or voiding or the pars libera during filling, marked by a line in **A**–**C**). The hashtag shows marked dilation of the posterior urethra (**D**)

There is consensus that if the posterior urethra is not dilated on VCUG (direct sign), an obstruction can be ruled out [12]. However, recent studies report that preoperative VCUG suspicion was present in only 46% of non-toilet-trained and 59% of toilet-trained males with PUV [11, 12]. The relatively low rate of PUV suspicion on VCUG may be due to the lack of identification of indirect signs of PUV (hypertrophied bladder neck, musculus interuretericus hypertrophy, and trabeculated appearance of the bladder wall) (Fig. 3). Only about 50% of patients with PUV exhibit direct signs on VCUG, with a sensitivity of 51.2% for PUV identification. When only indirect signs are considered, the sensitivity for PUV diagnosis is 58.1%. Notably, when both direct and indirect signs are taken into account, the sensitivity for diagnosing PUV rises to 97.7% and the negative predictive value rises to 98.5% [33]. Therefore, to optimize VCUG interpretation and reduce the percentage of “missed PUV” cases, it is crucial to recognize and value the indirect signs of PUV.

**Fig. 4:**
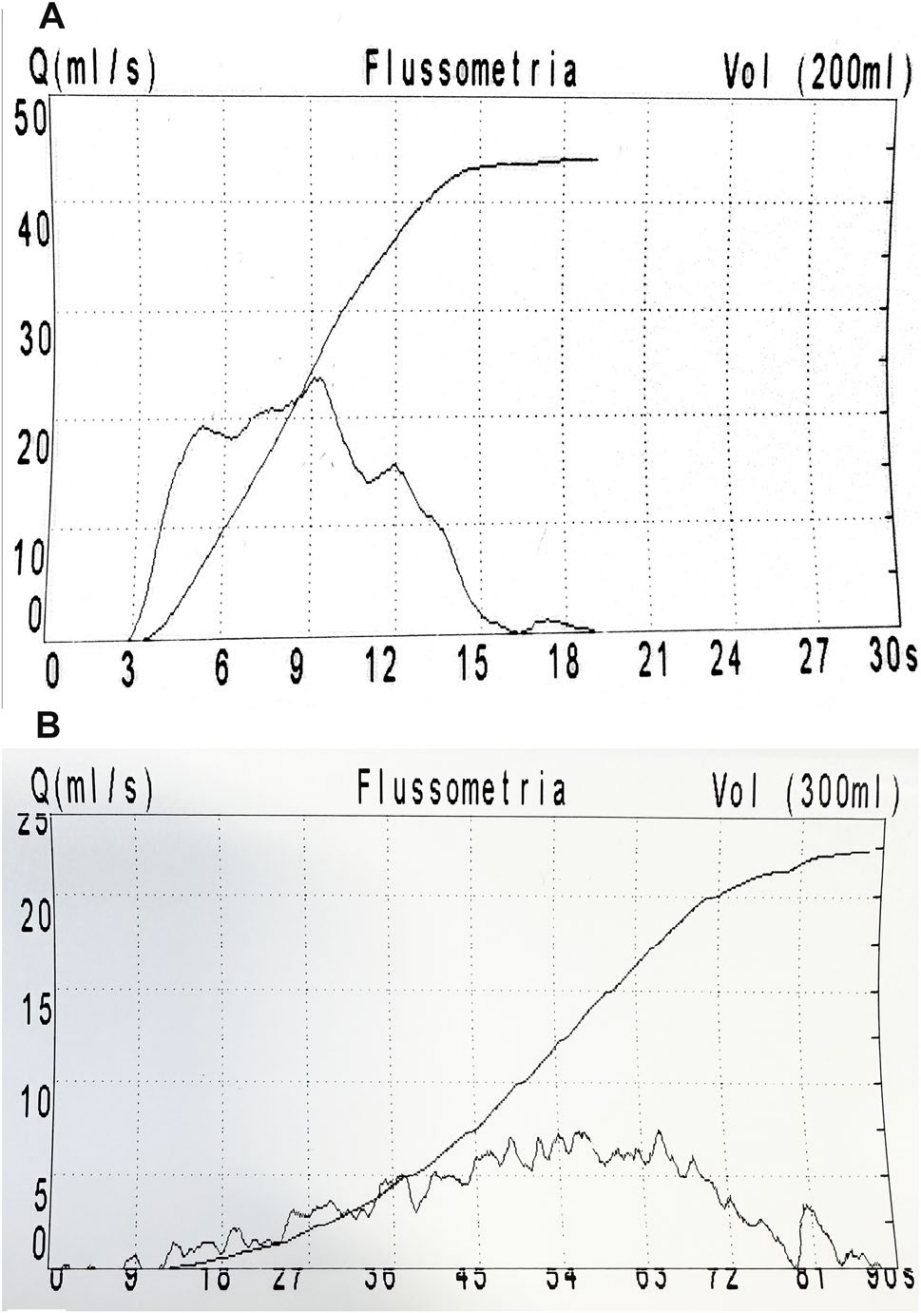
Uroflowmetry patterns. **A** Normal uroflowmetry pattern. **B** Abnormal uroflowmetry pattern suggesting PUV in an 8-year-old boy with micturition symptoms (urge-incontinence and occasional hesitancy to initiating micturition). The uroflowmetry reveals a plateau-shaped curve, a maximal flow of 7 mL/s (< 5th percentile), and a bladder voiding time of 70 s, suggesting the presence of infravesical obstruction, most likely due to PUV

However, although rarely [12], PUV can be detected on urethrocystoscopy despite the absence of direct and indirect radiological signs, but in the presence of persistent micturition symptoms and of an abnormal uroflowmetry pattern with reduced Q max (Fig. 4). Therefore, we wonder if, in selected cases of toilet-trained males with a strong suspicion of PUV, VCUG could be avoided and directly replaced by urethrocystoscopy. This would allow for the simultaneous diagnosis and treatment of a highly probable urethral obstruction, eliminating the need for a prior redundant invasive procedure [33]. Future clinical studies (focused on standardizing symptom assessment and repeated uroflowmetries) and improved local sonographic expertise (such as visualization of the urethra using perineal ultrasound without catheterization) should, in our opinion, aim to identify males with a strong suspicion of PUV who could undergo urethrocystoscopy without a prior VCUG.

CeVUS is also used for evaluating the male urethra [2]. Several studies have reported its feasibility in delineating urethral anatomy, and Damasio et al. advocate for the consideration and promotion of ceVUS whenever feasible, particularly due to its radiation protection benefits [2]. However, in cases with inconclusive ultrasound findings, VCUG remains necessary [2, 64]. Therefore, VCUG continues to be considered the radiological gold standard for assessing urethral anatomy and excluding PUV [2]. Additionally, challenges remain related to local expertise in using ceVUS and the inherent limitations of the technique, such as artifacts caused by intestinal gas that can mimic contrast microbubbles or posterior shadowing due to the presence of gas within the bladder, inability to obtain panoramic images of the urinary tract, and the learning curve [2].

In our opinion, however, it is essential to avoid misdiagnosing PUV as much as possible. A missed diagnosis of PUV, much more so than a missed diagnosis of VUR, can increase the risk of chronic kidney disease, particularly in neonatal PUV [65], and may also increase the risk of persistent micturition disorders in late PUV cases.

## Conclusions

Indications for VCUG are limited and should be guided by the potential clinical benefit of the diagnosis. VCUG for VUR diagnosis should be reserved for those with highly recurrent fUTIs (> 2 episodes, in our opinion), where surgical correction of a possible VUR could benefit the patient, and after thoroughly investigating, studying, and treating any potential bladder dysfunction.

In males, VCUG may be indicated in cases of fUTI with kidney dysplasia detected on ultrasound, as they are at risk of VUR as a “disease.” Nevertheless, even when VCUG is correctly performed, the possibility of occult VUR should be kept in mind. If recurrent fUTIs persist despite a negative VCUG and the absence of other causes, an alternative diagnostic technique with continuous monitoring of bladder filling and voiding, such as IC or ceVUS, may be warranted.

Additionally, careful attention should always be paid to radiation dose, following standardized protocols. In females, techniques without radiation, such as ceVUS, may be preferred as first-line options. In males, however, VCUG should be the preferred choice to obtain information about the urethra. Finally, it is crucial to maximize the information obtained from VCUG image interpretation, particularly regarding urethral morphology. When analyzing VCUG, indirect signs of PUV should be considered to improve diagnostic accuracy. In toilet-trained males, micturition symptoms and non-invasive urodynamics (uroflowmetry followed by ultrasound evaluation of post-void residual) should also be taken into account to raise suspicion of PUV. In selected cases, when suspicion of PUV is strong, first-line urethrocystoscopy may be considered.

## Key summary points

1. Voiding cystourethrography (VCUG) remains a key diagnostic tool for evaluating bladder and urethral anatomy and for diagnosing vesicoureteral reflux (VUR). Its limitations and pitfalls can be minimized through proper, standardized execution and accurate interpretation of images within the clinical context. Nevertheless, in a non-negligible proportion of cases, VUR may go undetected (“occult” VUR), and posterior urethral valves (PUV) may remain undiagnosed.
2. There is no clear consensus on the effectiveness of antibiotics in preventing post-procedural urinary tract infection (UTI). Regardless of its use, it is essential to inform parents about the potential risks and educate them on how to promptly recognize signs and symptoms of a UTI.
3. In cases of suspected occult VUR—where VCUG is negative despite highly recurrent UTIs and no evidence of bladder dysfunction—it may be reasonable to investigate VUR using a more sensitive method, such as isotopic cystography or contrast-enhanced voiding urosonography.
4. If the posterior urethra is not dilated and no indirect signs—such as a hypertrophied bladder neck, musculus interuretericus hypertrophy, or bladder wall trabeculation—are visible on VCUG, urethral obstruction can generally be ruled out. However, in cases of strong clinical suspicion of undiagnosed urethral obstruction in males (i.e., persistent micturition symptoms and abnormal uroflowmetry despite no direct or indirect signs on VCUG), cystourethroscopy may still be warranted. In selected cases with high clinical suspicion, it may be reasonable to proceed directly to cystourethroscopy without performing VCUG, thereby reducing radiation exposure and discomfort for the child.

## Multiple choice questions

Answers are given following the references.

1. When is VCUG indicated?
  - Recurrent febrile UTIs
  - Abnormal uroflowmetry and/or symptoms suggesting infravesical obstruction in males
  - Mono- or bilateral megaureter>7mm of diameter at post-natal KUS with fUTI
  - All of the above
2. Which is the correct answer regarding VCUG procedure?
  - It is not necessary to use a clean procedure for catheterization
  - Lidocaine urethral gel can be used as both an anesthetic and a lubricant
  - Sedation is mandatory
  - Foley catheter is required for procedure
3. Find the correct statement on VCUG when used to detect VUR
  - VCUG cannot miss VUR diagnosis
  - VCUG is more sensitive than isotopic cystography for VUR
  - When VUR is detected, an antibiotic prophylaxis must be prescribed regardless of VUR grade
  - None of the above
4. What of the following sentences are correct?
  - Presence of urethral dilation on VCUG should rise the suspect of PUV
  - The absence of direct and indirect signs rules out PUV with negative predictive value of 98.5%
  - PUV can be detected on urethrocystoscopy despite the absence of direct and indirect radiological signs
  - All of the above

## Supplementary information

Below is the link to the electronic supplementary material.

Graphical abstract (PPTX 80.1 KB)

## Data Availability

Not applicable.

## Abbreviations

*ALARA*: as low as reasonably achievable
*AP*: anteroposterior
*VCUG*: voiding cystourethrography
*UTI*: urinary tract infection
*VUR*: vesicoureteral reflux
