# Supplementary material for: Voiding cystourethrography for the pediatric nephrologist: clinical value, challenges, and areas of debate
Source: Pediatr Nephrol. 2025 Sep 9;41(5):1287–301. doi: 10.1007/s00467-025-06901-3 (PMC13009064; doi:10.1007/s00467-025-06901-3)
Supplement: Supplementary file 1 — Graphical abstract (PPTX 80.1 KB) [file 467_2025_6901_MOESM1_ESM.pptx]

## Slide 1
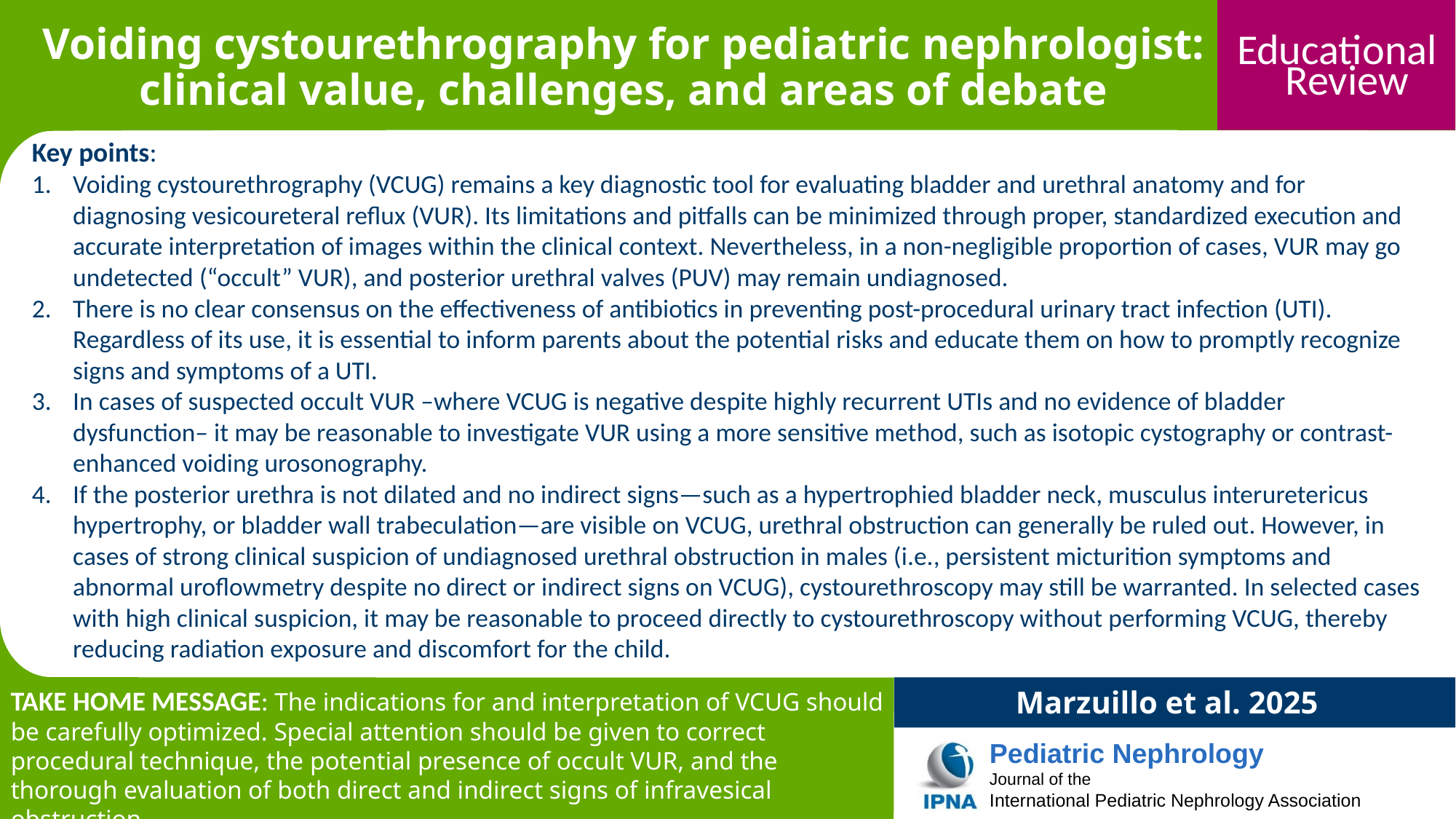

Voiding cystourethrography for pediatric nephrologist: clinical value, challenges, and areas of debate
Key points:
Voiding cystourethrography (VCUG) remains a key diagnostic tool for evaluating bladder and urethral anatomy and for diagnosing vesicoureteral reflux (VUR). Its limitations and pitfalls can be minimized through proper, standardized execution and accurate interpretation of images within the clinical context. Nevertheless, in a non-negligible proportion of cases, VUR may go undetected (“occult” VUR), and posterior urethral valves (PUV) may remain undiagnosed.
There is no clear consensus on the effectiveness of antibiotics in preventing post-procedural urinary tract infection (UTI). Regardless of its use, it is essential to inform parents about the potential risks and educate them on how to promptly recognize signs and symptoms of a UTI.
In cases of suspected occult VUR –where VCUG is negative despite highly recurrent UTIs and no evidence of bladder dysfunction– it may be reasonable to investigate VUR using a more sensitive method, such as isotopic cystography or contrast-enhanced voiding urosonography.
If the posterior urethra is not dilated and no indirect signs—such as a hypertrophied bladder neck, musculus interuretericus hypertrophy, or bladder wall trabeculation—are visible on VCUG, urethral obstruction can generally be ruled out. However, in cases of strong clinical suspicion of undiagnosed urethral obstruction in males (i.e., persistent micturition symptoms and abnormal uroflowmetry despite no direct or indirect signs on VCUG), cystourethroscopy may still be warranted. In selected cases with high clinical suspicion, it may be reasonable to proceed directly to cystourethroscopy without performing VCUG, thereby reducing radiation exposure and discomfort for the child.
Marzuillo et al. 2025
TAKE HOME MESSAGE: The indications for and interpretation of VCUG should be carefully optimized. Special attention should be given to correct procedural technique, the potential presence of occult VUR, and the thorough evaluation of both direct and indirect signs of infravesical obstruction.
